# Cytomegalovirus enteritis resistant to antiviral drugs improved following total colectomy

**DOI:** 10.1186/s40792-023-01672-1

**Published:** 2023-06-15

**Authors:** Sae Kawata, Jumpei Takamatsu, Yuichi Yasue, Aya Fukuhara, Jinkoo Kang

**Affiliations:** Department of Emergency Medicine, Kansai Rosai Hospital, 3-1-69 Inabaso, Amagasaki, Hyogo 660-0064 Japan

**Keywords:** Cytomegalovirus enteritis, Surgical therapy, Antiviral drug resistance

## Abstract

**Background:**

Cytomegalovirus (CMV) infection, often subclinical in childhood, is reactivated during a state of cell-mediated immunodeficiency. In cases of organ damage, patients can require medical treatment for an infectious disease, generally through the use of antiviral drugs. There are no reports of surgical treatment in cases, where infection was found, and medical treatment was difficult. We encountered a case of CMV enteritis that was difficult to treat because of resistance to antivirals but improved after total colectomy.

**Case presentation:**

A previously healthy, 74-year-old woman visited a doctor with a chief complaint of watery diarrhea persisting for 2 weeks; she was transferred to our hospital because of hypoxemia and hypovolemic shock. Computed tomography scan indicated wall thickening over the entire colon and the patient was diagnosed with infectious colitis. Conservative and antibacterial therapies were started with fasting fluid replacement. Subsequently, bloody stools were observed 11 days after admission. Colonoscopy was then performed, which showed mucosal edema and longitudinal ulcer, while a histopathological examination of the colon mucosa revealed C7HRP positive on 22 days after admission. CMV enteritis was diagnosed, and the antiviral medication, ganciclovir, was started. Diseases causing immunosuppression and other possible causes of enteritis were also closely examined; however, all were negative. Furthermore, the patient’s symptoms and her endoscopic findings did not improve with ganciclovir administration; therefore, the antiviral drug was changed to foscarnet. Unfortunately, the patient did not improve despite the additional administration of gamma globulin and methylprednisolone, and she was determined to have enteritis resistant to medical therapy. A total colon resection was performed 88 days after the admission. Her condition gradually stabilized postoperatively, and oral intake was initiated and tolerated. The patient was transferred to another hospital for rehabilitation for home discharge. She is now at home and has had no recurrences.

**Conclusions:**

In previous reports of surgical treatment for CMV enteritis, many cases were initially undiagnosed, emergency surgery was performed after perforation or stenosis was recognized, and then CMV was diagnosed and treated. In CMV enteritis without immunodeficiency, surgical treatment may be an option if medical treatment is ineffective.

## Background

Cytomegalovirus (CMV) has been well-recognized as a pathogen which causes opportunistic infections in immunocompromised patients [1, 2]. After primary infection, a latent virus can reactivate in immunosuppressed states, such as stem cell or solid organ transplantation, autoimmune diseases, or acquired immunodeficiency syndrome (AIDS) [1, 3, 4]. CMV diseases following reactivation include pneumonitis, enteritis/colitis, retinitis, and hepatitis [3].

CMV infections are regularly treated with antiviral drugs, such as ganciclovir or foscarnet [5]. The duration of treatment is unclear, but CMV is generally cured after 2–3 weeks of drug administration [6]. However, with CMV enteritis, statistical reports in Japan indicate that 62% of cases involving perforation cause death [7]. Moreover, 14.3% of immunocompromised CMV enteritis cases relapse, and 12.5% cause death [8]. With many individuals infected and treated in an immunocompromised state, the disease is often considered severe if medical treatment is ineffective.

We hypothesize that if clinicians are unaware of immunodeficiency, surgical treatment can be considered an option in cases of refractory disease. Most of the previous reports involving surgical treatment for CMV enteritis and infection indicated that the cases were diagnosed and treated after emergency surgery for perforation or stenosis [9–12]. This is the first time that surgical treatment was performed, because infection was found, and medical treatment was difficult. Therefore, we report a case of CMV enteritis that was difficult to treat due to antiviral medication resistance but improved after performing a total colectomy.

## Case presentation

**Fig. 1:**
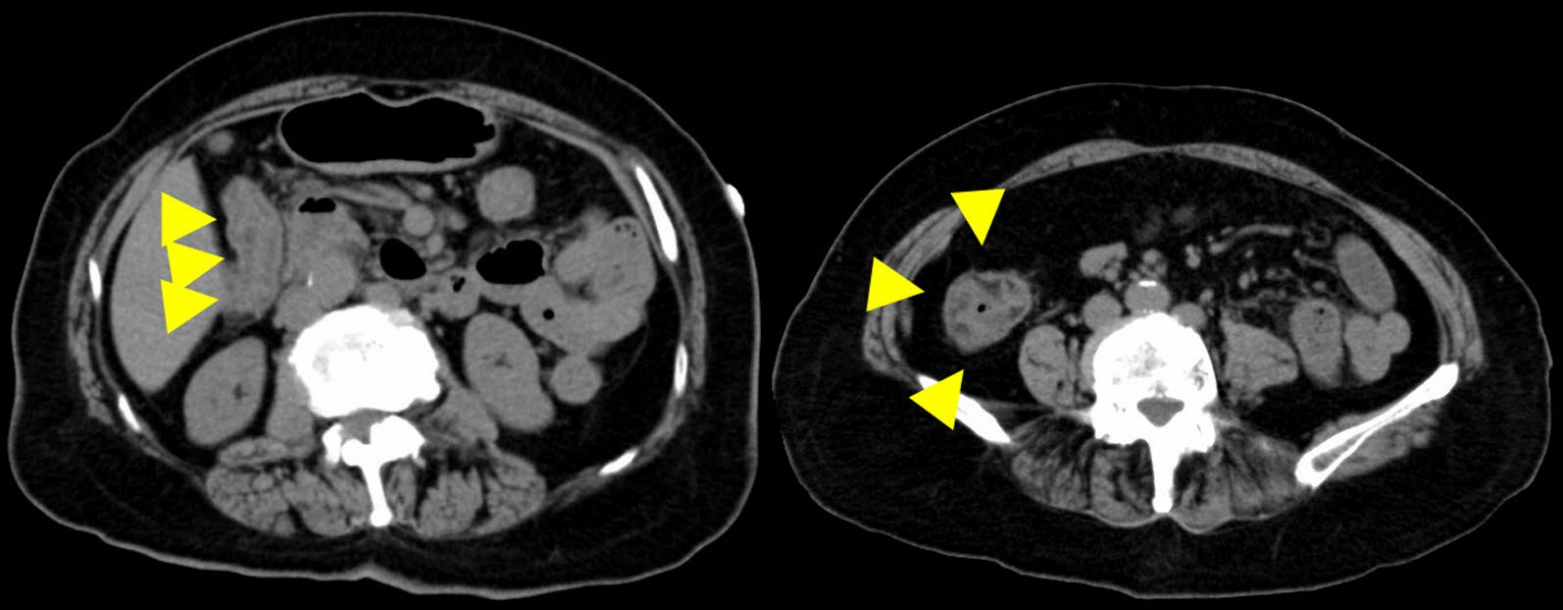
Chest-abdominal CT. Chest-abdominal CT examination showed edema of the duodenum and wall thickening in the entire colon, especially in the ascending to transverse colons

A 75-year-old woman without an immunodeficiency history was referred to our hospital because of hypoxemia and shock. She visited a former doctor with a complaint of watery diarrhea that had persisted for 10 days and was diagnosed and treated for acute gastroenteritis. Her medical history included chronic atrial fibrillation and cardiogenic cerebral infarction. During her visit at our hospital, she had clear consciousness of the Glasgow Coma Scale E4V5M6, presented hypoxemia with SpO_2_ 97% under oxygen 12 L/min, respirations 20/min, shock with a blood pressure of 70/40 mmHg, heart rate of 91 beats/min, a respiratory rate of 23 breaths/min, body temperature of 36.3 °C, and tenderness throughout the abdomen. Her height was 151 cm, weight was 59.8 kg, while his body mass index (BMI) was 26.2 kg/m^2^. Laboratory data revealed increased white blood cells (14.9 × 10^9^/L), increased C-reactive protein (18.8 mg/dL), acute kidney injury (blood urea nitrogen level: 52.6 mg/dL; creatinine level: 4.35 mg/dL), coagulopathy (international normalized ratio [INR]: 2.3; activated partial thromboplastin time [aPTT]: 48.9 s; d-dimers 15 μg/mL), and hypoproteinemia (albumin: 2.5 g/dL; total protein: 5.5 g/dL). A whole-body computed tomography (CT) scan without a contrast agent showed edema of the duodenum, and wall thickening was observed in the entire colon, especially from the ascending to the transverse colon (Fig. 1). Thus, the patient was diagnosed with general infectious colitis and started on conservative and antibacterial therapies with fasting fluid replacement. However, abdominal pain and diarrhea intensified, and the CT scan performed also showed worsening edema of the colon and marked intestinal dilatation. In addition, the patient had atrial fibrillation and required rehydration for severe diarrhea, which made volume control difficult. Subsequently, the patient's respiratory condition deteriorated due to complications, such as heart failure and pulmonary edema, which required intubation and ventilatory management.

**Fig. 2:**
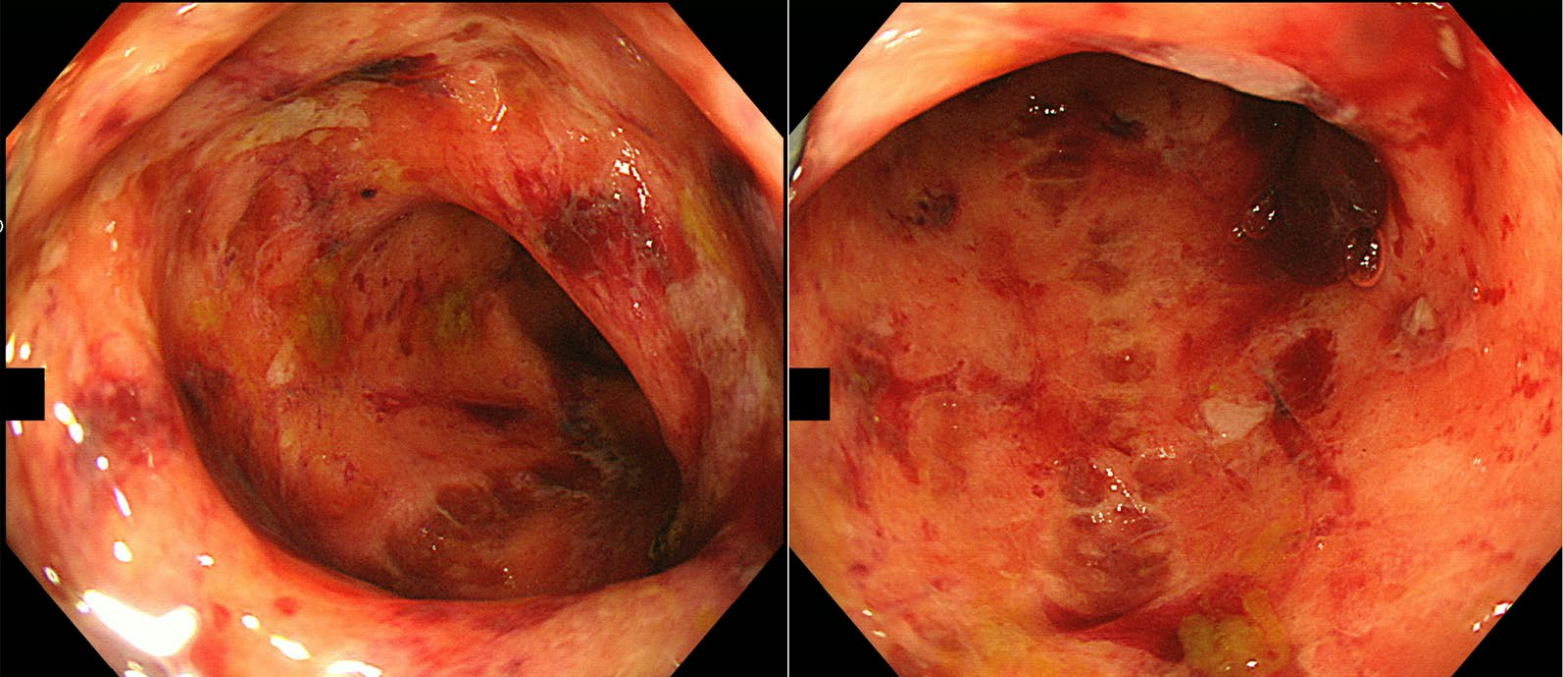
Colonoscopy performed on day 19. Colonoscopy showed mucosal edema in the sigmoid colon. Easy bleeding throughout sigmoid colon, slightly deep longitudinal ulcer. There was nothing abnormal in the rectum

**Fig. 3:**
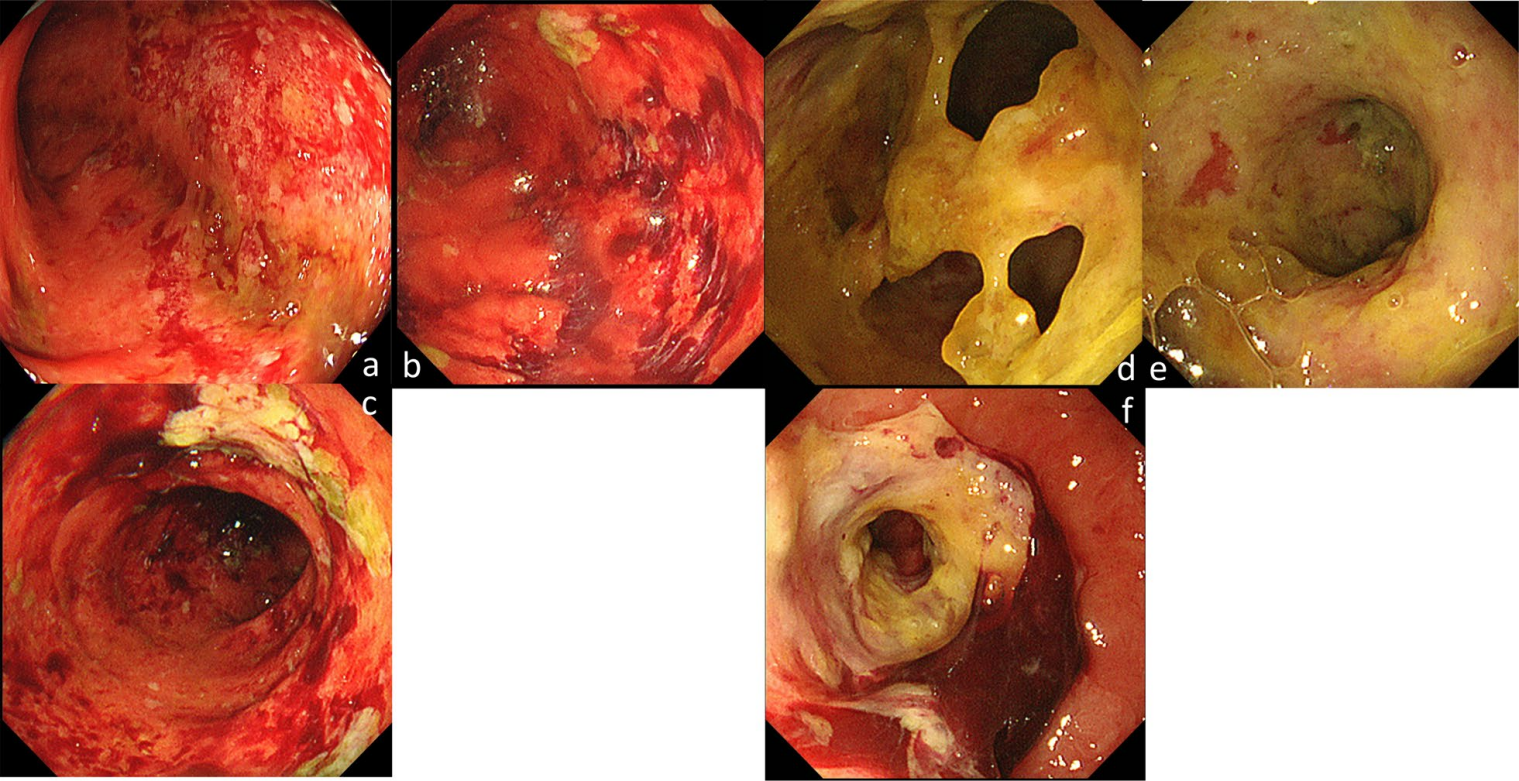
Colonoscopy performed on day 40, 75. Colonoscopy performed on day 40 (**a**–**c**) and day 75 (**d**–**f**) after admission. The colon is edematous throughout, with scattered erythema and erosions (**a**). The sigmoid colon has particularly strong inflammatory changes, with scattered longitudinal ulcers. Slight improvement from the previous colonoscopy (**b**, **c**). A circumferential ulcer and stricture are noted 2 cm from the antral dentate line (**f**). Beyond the stricture is a fairly wide lumen with necrotic material in the mucosa or rectal wall (**d**, **e**). Perforation of the retroperitoneum was a highly suspicious finding. On its mouth side, a circumferential ulceration continued

**Fig. 4:**
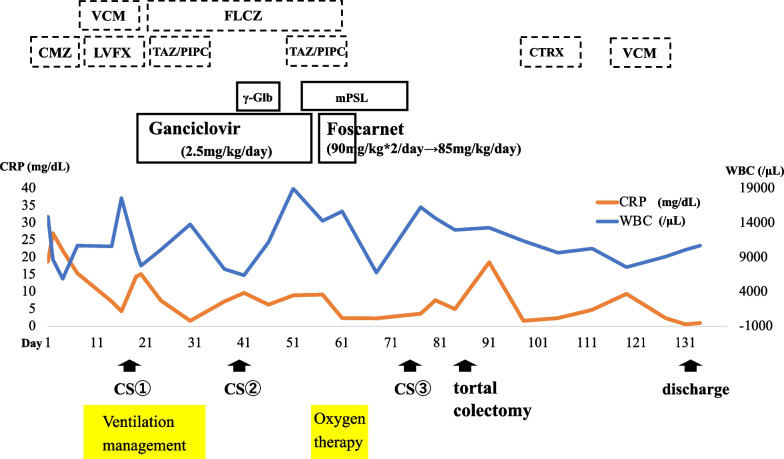
Clinical course. CS ①: colonoscopy performed on the 19th day (Fig. 2), CS ②: colonoscopy performed on the 40th day (Fig. 3), CS ③: colonoscopy performed on the 75th day (Fig. 3). *VCM* vancomycin, *FLCZ* fluconazole, *CMZ* cefmetazole, *LVFX* levofloxacin, *TAZ/PIPC* tazobactam/piperacillin, *CTRX* ceftriaxone, *CS* colonoscopy

Bloody stools were observed on day 11 after admission, while a colonoscopy performed on day 19 showed a generally edematous mucosa; the sigmoid colon was frequently hemorrhagic, with a few deep-drilled longitudinal ulcers (Fig. 2). The inflammation was so severe that we could not perform a total colonoscopy. The distribution and appearance of the lesions were not endoscopic findings highly suspicious of inflammatory bowel diseases, such as ulcerative colitis (UC) or Crohn's disease. Histopathological examination of the colon mucosa revealed positive CMV antigenemia (C7HRP) day 22 after admission. Ganciclovir (2.5 mg/kg/day) was started on day 22 when CMV infection was strongly suspected. The patient was found to have serum CMV–deoxyribonucleic acid (DNA) positive, CMV–immunoglobulin (Ig) G 3.7 (EIA cutoff < 1.9), and CMV–IgM 0.26 (EIA cut off < 0.79). In addition to the symptoms and endoscopic findings, the diagnosis of CMV enteritis was considered definite. Mucosal histology at the time of endoscopy showed no findings suggestive of infection by other pathogens or inflammatory bowel disease. Diseases causing immunosuppression and other possible causes of enteritis were also closely examined (Table 1); however, all were negative. After ganciclovir was started, hematochezia disappeared temporarily, but gastrointestinal symptoms, such as diarrhea and abdominal pain did not improve. On day 40, colonoscopy was performed, and the mucosa of the large intestine was generally edematous with redness and erosion. Inflammatory changes were particularly worse in the sigmoid colon, longitudinal ulcers and round ulcers were scattered, and the condition was slightly worse than the previous time (Fig. 3). Gamma-globulin 5 g/day volume for 3 days was administered for 3 days. In addition, ganciclovir was administered for 33 days, but the patient's condition worsened; thus, she was considered to have ganciclovir-resistant CMV, and antiviral treatment was changed from ganciclovir to foscarnet on day 55. Foscarnet was started at a volume of 90 mg/kg twice daily, but due to strong side effects of renal dysfunction and electrolyte abnormalities, the dose was reduced to 85 mg/kg once daily and continued. However, there was no progress even with foscarnet, and the treatment was terminated after 11 days because of drug-induced renal injury. Moreover, she had persistent abdominal pain and watery stool. She was considered to have other complications and was treated with methylprednisolone (125 mg/day for 3 days, 40 mg/day for 7 days, 30 mg/day for 7 days); Due to worsening symptoms, methylprednisolone was tapered off early. In addition, the patient had a large number of watery stools, difficulty in volume control, pleural effusion, and temporary respiratory failure (Fig. 4). Colonoscopy was performed on day 75 of hospitalization and revealed a full circumferential ulcer in the rectum, which was highly suspicious for mucosal desquamation and perforation into the retroperitoneum.

**Table 1:** Laboratory test of immunology

Item	Result	Unit	Item	Result	Unit
HIV	Negative		Anti-nuclear antibody	80	X
IL-2R	2693.9	U/mL	Anti-ds DNA IG	Negative	
IgG	568	mg/dL	Anti-SS-A	Negative	
IgA	181	mg/dL	Anti-SS-B	Negative	
IgM	29	mg/dL	Anti-CCP	< 0.6	U/mL
HTLV-1	Negative		PR3–ANCA	< 1.0	U/mL
CD3	86.8	%	MPO–ANCA	< 1.0	U/mL
CD4	66	%			
CD3/4	2.53	%

**Fig. 5:**
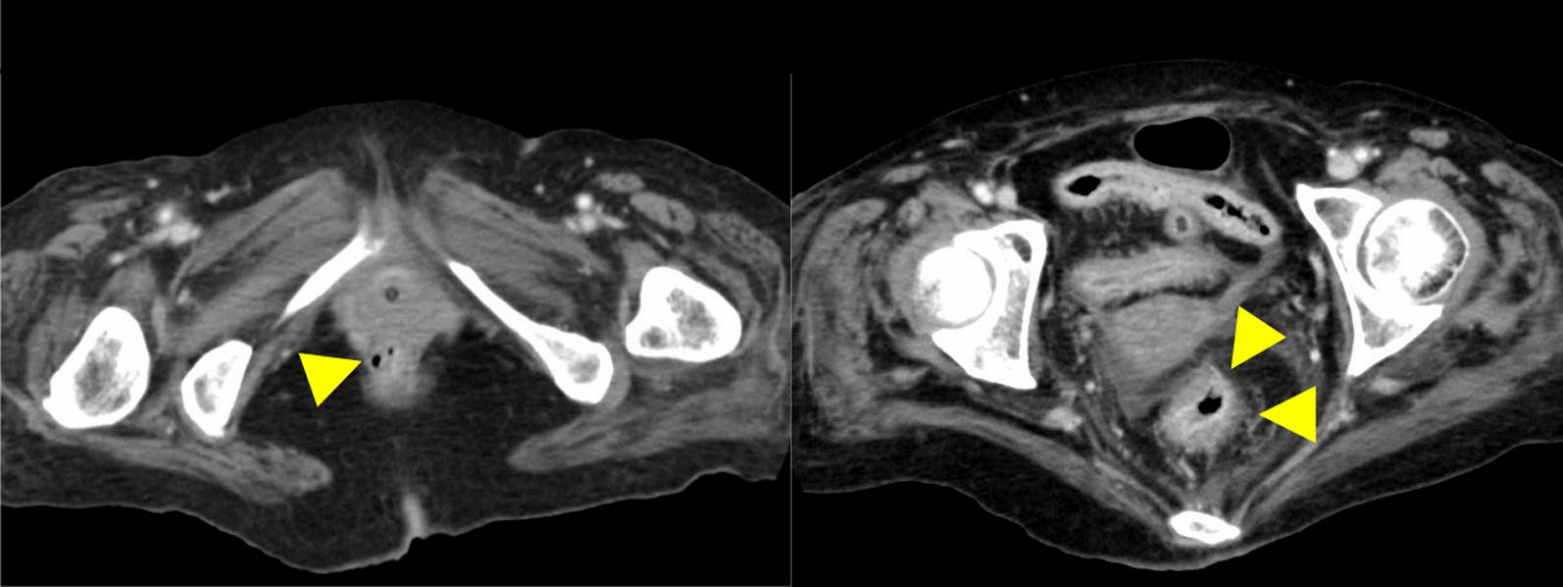
Abdominal contrast-enhanced CT. The rectum showed edematous wall thickening throughout. Some of the findings are thinning. Perforation into the abdominal cavity was thought to be only a matter of time

**Fig. 6:**
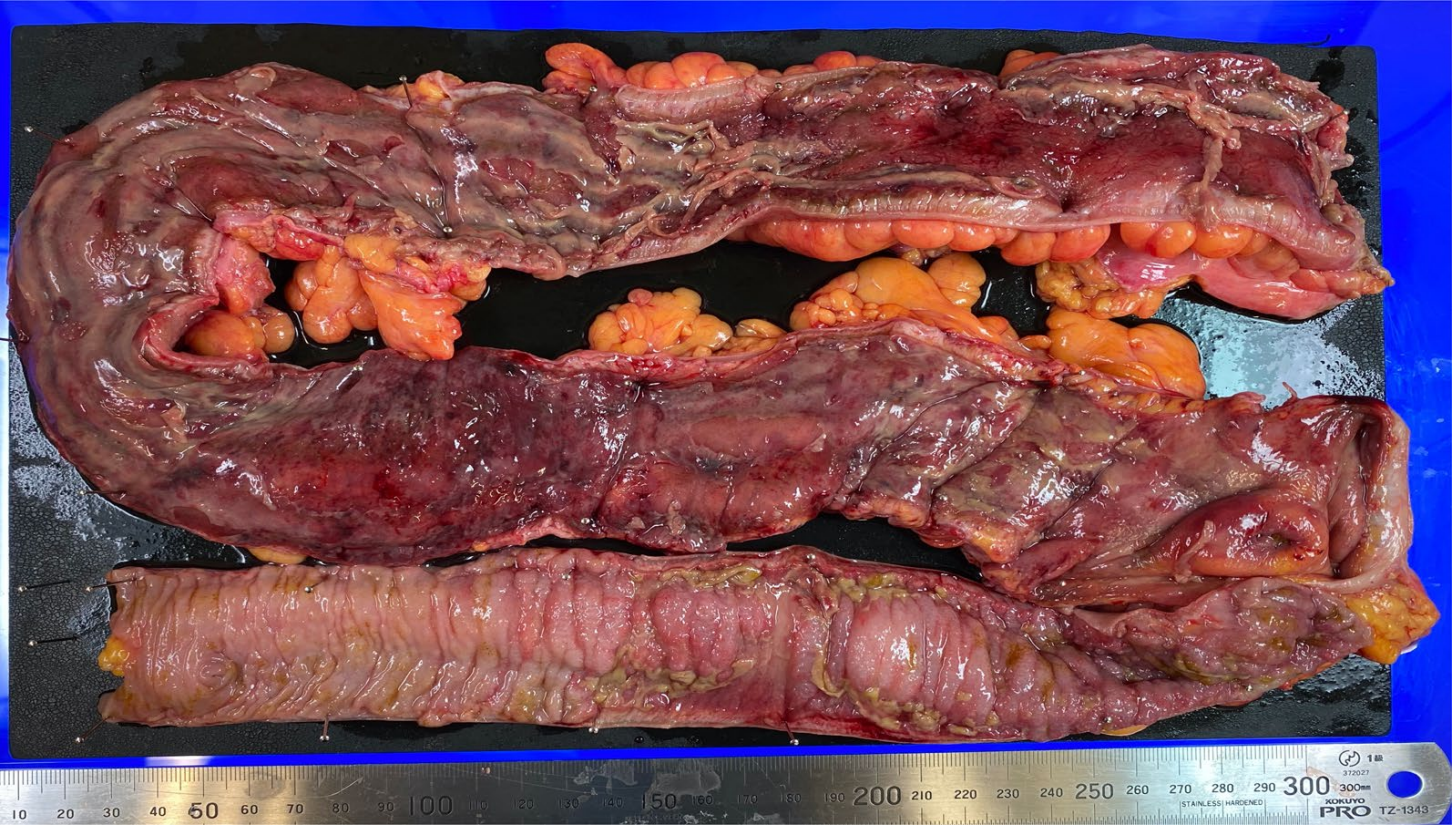
Intestinal resection specimen. Total colectomy, partial resection of the small intestine (50 cm), and monotreme colostomy were performed on day 88 after admission. The resected specimen shows whole circumferential mucosal desquamation of the sigmoid to descending colon and scattered mucosal dissection of the transverse to ascending colon. Longitudinal ulcers are observed in the small intestine

**Fig. 7:**
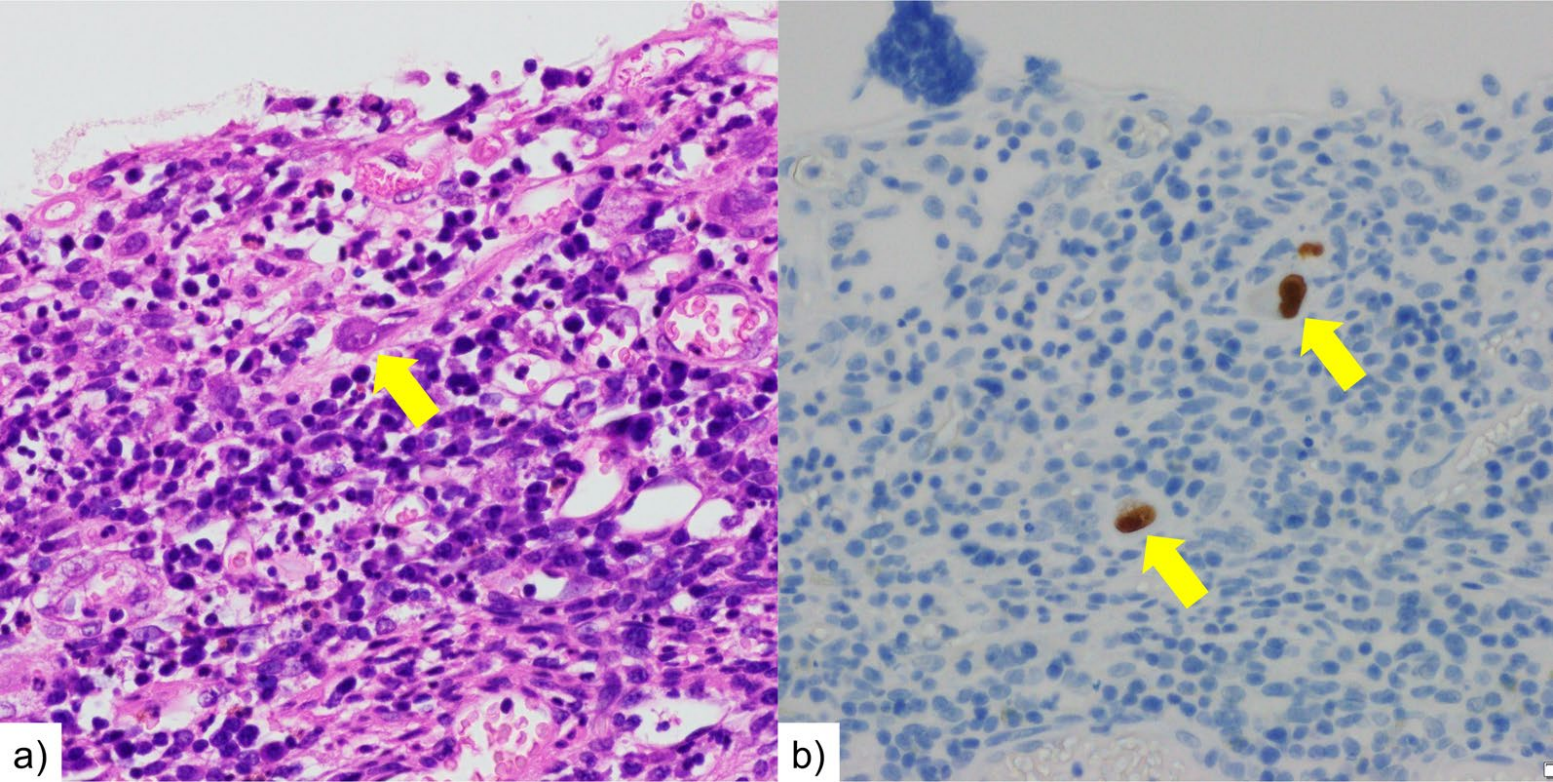
Histopathological examination results of resected intestinal specimens. HE staining 400 × (**a**), CMV immunostaining 400 × (**b**). Pathology shows extensive necrosis and degeneration of the mucosal epithelium, and the mucosal intrinsic layer was replaced by granulation tissue and fibrin with a high inflammatory cell infiltrate. HE staining (**a**) and CMV immunostaining (**b**) show nuclear inclusion bodies, indicating CMV infection. There is no evidence of inflammatory bowel disease, granuloma, or vasculitis

Medical treatment was ineffective, and the gastrointestinal findings continued to worsen over time. In addition, colonoscopy showed rectal mucosal shedding and perforation of the retroperitoneum. Initially, the patient required ventilation and intensive care management. Once his condition stabilized and he was discharged from the intensive care unit, he again developed respiratory failure due to poor volume control and required intensive care and oxygen therapy. However, although she was undernourished, her state of consciousness was clear and had stable circulation and inflammation; thus, we decided to perform surgical interventions. Colonoscopy and CT scan revealed that the rectum was in communication or nearly in communication with the retroperitoneal free space, and edema in the entire large intestine and section of the small intestine (Fig. 5). It was difficult to assess the extent of the infected intestinal tract and to determine the extent of surgical bowel resection. Preoperative contrast-enhanced CT scan confirmed the extent of small intestinal mucosal edema. The small intestine 40–50 cm from the ileum to the mouth showed continuous wall thickening, which was determined to be an infected intestinal tract. It was decided to perform a combined resection of the small intestine with edema. Intraoperative endoscopy was also prepared. On day 88, total colectomy, partial resection of the small intestine (50 cm), and monotreme colostomy were performed. The intestinal serosa showed no gross findings of ischemia or inflammation. The colon and small intestine showed strong adhesions that may have been due to inflammatory changes. Rectal resection was performed just above the peritoneal reflection. The small intestine was manually checked and resected, where the mucosal edema seemed particularly strong. Intraoperatively, the resection specimen was viewed to confirm that the small bowel resection margins were not highly inflamed. The excised intestinal tract showed detachment of the entire mucous membranes (Fig. 6). The resected intestinal specimen histopathological examination revealed inflammatory granulation of the tissue with necrosis and a high lymphocyte-predominant inflammatory cell infiltration. Hematoxylin and eosin (HE) staining and CMV immunostaining showed nuclear inclusion bodies, indicating CMV infection (Fig. 7). Histopathological examination did not reveal any other causes of enteritis. Her general condition improved postoperatively, and oral intake resumed. Serum CMV DNA was negative on day 119. She was then transferred to a transitional care facility for rehabilitation on day 139 and was discharged on day 582. She is now at home and has had no recurrences. Activities of Daily Living is wheelchair-bound and can transfer to a wheelchair by herself. He is able to manage his colostomy by herself and has no dementia. Home health care is intervening, but food intake is good and no supplemental fluids are being administered. Home nursing care is used once a week.

## Discussion

CMV is first detected in early childhood and is latent in life [1, 3, 4]. Most are initially subclinical infections and are often reactivated in immunosuppressed conditions [2]. In Japan, ~ 80–90% of people are infected with CMV in childhood [13]. The risk factors for reactivation are immunodeficiency, steroid use, shock, antibacterial agent administration, radiation therapy, and chronic kidney disease [13]. In recent years, there have been reports of disease development in older patients who do not have these underlying risk factors [3, 12]. Of the 16 cases of CMV enteritis without serious illness or prior immunosuppressive treatment reported to date, 10 were older than 65 years of age. Of those 10 cases, only one case recovered spontaneously, and four cases eventually required surgery [14]. A meta-analysis of 44 cases of CMV enteritis in healthy adults, analyzed retrospectively, found that only 31.8% of cases resolved spontaneously, and when further stratified by age, half of the cases in patients younger than 55 years achieved spontaneous resolution, compared to a low rate of 25% in patients over 55 years [15]. These reports suggest that spontaneous recovery of CMV enteritis in healthy elderly patients is less common than previously thought, and that surgical treatment is often necessary even after medical treatment, such as antiviral therapy.

Treatment of CMV infection typically involves reduction of immunosuppressants in immunocompromised patients and treatment with antiviral drugs, such as ganciclovir and foscarnet. In immunocompetent patients, CMV infection is often resolved by an autoimmune response. The optimal duration of antiviral therapy has not yet been established. Treatment for at least 2 weeks is required, and when clinical symptoms improve or viremia is present, polymerase chain reaction (PCR) or C7HRP is confirmed to be negative twice consecutively before treatment termination [5]. In general, the treatment course is 2–3 weeks [6]. For most patients, treatment involved the administration of anti-CMV agents (ganciclovir, valganciclovir, and foscarnet). However, in recent years, cases wherein treatment resistance developed even after administration of ganciclovir have been reported [16, 17]. In such cases, increasing the dose of ganciclovir or switching to foscarnet is considered.

Surgical intervention is a difficult option when the medical treatment is refractory. Infectious enteritis is treated with symptomatic therapy, including fluid replacement as well as antimicrobial and antiviral drugs. In most of the reported cases of surgical treatment for CMV enteritis, emergency surgery was unavoidable due to perforation or bleeding and there was no prior medical treatment; the diagnosis was confirmed after the surgery [9–12]. Seven case reports were detected when searching for CMV enteritis and Surgery, in the Central Journal of Medicine and PubMed for the 5-year period 2018–2023 (Table 2) [18–24]. Two of these cases were diagnosed as CMV enteritis and medical treatment was initiated, but they soon perforated, resulting in emergency surgery [22, 23]. The remaining five cases were not diagnosed preoperatively and were operated on urgently. All cases were emergency surgeries with the expectation of bleeding or perforation. We could not find any cases that showed resistance to medical treatment or wherein surgical treatment was chosen after being diagnosed with CMV infection. This could be attributed to the fact that anti-CMV drugs are basically effective and patients with treatment-resistant-CMV infection may be immunocompromised as an underlying disease, which makes them unfit for surgery owing to poor health.

**Table 2:** Surgical case reports for CMV

No.	Years	Author	Age	Sex	Chief complaint	Preoperative diagnosis	Surgical treatment
1	2018	Takamura	33	M	bloody stool	Ischemic colitis	Left hemicolectomy, colostomy
2	2019	Komatsuzaki	62	M	Fever	Perforated appendicitis	Ileocecal resection
3	2019	Higuchi	69	M	Stomach ache	Toxic megacolon	Subtotal colectomy, colostomy
4	2020	Takayama	57	M	Bloody stool	Jejunal bleeding	Partial small bowel resection
5	2020	Song	39	M	Stomach ache	Intestinal perforation	Right hemicolectomy, partial small bowel resection, colostomy
6	2021	Wang	28	M	Bloody stool	Intestinal perforation	Partial small bowel resection
7	2022	Waisayarat	6	F	Stomach ache	Intestinal perforation	Partial small bowel resection

Searched on CMV enteritis and Surgery, in the Central Journal of Medicine and PubMed for the 5-year period 2018–2023

Inflammatory bowel disease is similar to infectious enteritis. In addition, Crohn's disease and UC are representative diseases, both of which are first treated by drugs, and when unsuccessful, surgical intervention is performed. In both cases, the cause of the disease is unclear, and medical treatment is often inadequate to control the disease, including symptoms. The absolute indications for surgery for both Crohn's disease and UC are colonic perforation, massive hemorrhage, toxic megacolon, and fulminant UC [25]. Although not emergent, relative surgical indications include refractory cases, stenosis, and fistula formation [25]. Refractory cases are defined as those wherein medical treatment is not effective, and patients experience difficulty in daily and social life, or have poor quality of life. The incidence of these two diseases is increasing, along with the number of cases involving surgical treatments. However, these have a higher perioperative complication rate than general bowel resection due to prolonged intestinal inflammation, low nutritional status, use of steroids and immunosuppressive drugs, and disease-specific ecologic abnormal reactions [26–28]. Crohn's disease, in particular, is characterized by skip lesions, and there is a risk of recurrence in the residual intestinal tract after surgery. This is also true in cases of CMV infection. It is a systemic infectious disease, and the possibility remains that the infected intestinal tract cannot be fully evaluated by CT or endoscopy [25].

In this case, there was no factor causing immune-compromising disease. In addition, no other diseases caused gastrointestinal ulcers (such as Crohn's disease or UC). Ganciclovir and foscarnet were administered as anti-CMV agents; however, they were ineffective, and the gastrointestinal symptoms of abdominal pain and diarrhea persisted. Colonoscopy also showed that the extent of inflammation increased with each passing day, and eventually, the rectal mucosa became necrotic and fused. Malabsorption of nutrients from the intestinal tract occurred due to prolonged fasting and massive diarrhea after starting food. Before the surgery, the patient was markedly undernourished. In addition, the patient had difficulty maintaining the fluid due to frequent diarrhea and low nutritional status, thus, making it difficult to manage arrhythmia and heart failure. Medical treatment failed to control the infection, and based on the colonoscopy findings, we thought perforation was inevitable; therefore, we performed surgery (total colectomy, partial resection of the small intestine, and monotreme colostomy). In this case, a third colonoscopy was barely performed before perforation, and the patient was saved from fatal peritonitis. Finally, the patient did not exhibit any symptoms and progressed well.

Symptomatic and medical therapy is the main treatment for generalized infectious enteritis. Surgical treatment for infectious enteritis is uncommon, typically only for toxic megacolon use. To the best of our knowledge, this is the first case to report a patient who was refractory even with adequate medical treatment in the CMV enteritis. Fasted management of older patients, even if not immunocompromised, facilitates poor activities of daily living and malnutrition, greatly affecting patient outcomes. Furthermore, in this case, cardiac dysfunction was initially recognized but continued infusion aggravated heart failure. These problems can pose risks when surgery is performed, while longer hospital stays increases the hospital bill. The first choice of treatment is the administration of anti-CMV agents and examination and treatment of the cause of immunosuppression; however, if symptoms worsen, regular evaluations, such as colonoscopy and CT should be performed at close intervals. When exacerbation is observed after repeated treatments, recovery after treating with medications/therapies cannot be expected. Ultimately, we believe that treatment methods should be reconsidered, because surgical treatment may cure the disease. In our case, had we made the decision to operate a little earlier, the patient might have recovered more quickly without suffering from complications, such as heart failure.

## Conclusions

CMV enteritis is common in immunocompromised patients and medical treatment is the standard of care. Many reported cases who underwent surgical treatment for CMV enteritis have not been diagnosed. In CMV enteritis without immunodeficiency, as in this case, if medical treatment is not effective, surgery may be an alternative.

## Data Availability

All data generated or analyzed during this study are included in this published article.

## Abbreviations

CMV: Cytomegalovirus
C7HRP: CMV antigenemia
aPTT: Activated partial thromboplastin time
CT: Computed tomography
UC: Ulcerative colitis
DNA: Deoxyribonucleic acid
Ig: Immunoglobulin
PCR: Polymerase chain reaction
